# Lipid-based nanoparticles: innovations in ocular drug delivery

**DOI:** 10.3389/fmolb.2024.1421959

**Published:** 2024-09-17

**Authors:** Mirza Salman Baig, Shweta Kulkarni Karade, Anas Ahmad, Mohd. Ashif Khan, Anzarul Haque, Thomas J. Webster, Md. Faiyazuddin, Noora H. Al-Qahtani

**Affiliations:** ^1^ Anjuman-I-Islam’s Kalsekar Technical Campus School of Pharmacy, Affiliated to the University of Mumbai, New Panvel, Maharashtra, India; ^2^ DY Patil Deemed to be University- School of Pharmacy, Navi Mumbai, India; ^3^ Department of Microbiology, Immunology and Infectious Diseases, Cumming School of Medicine, University of Calgary, Alberta, Canada; ^4^ Centre for Translational and Clinical Research, School of Chemical and Life Sciences, Jamia Hamdard, New Delhi, India; ^5^ Central Laboratories Unit (CLU), Qatar University, Doha, Qatar; ^6^ School of Health Science and Biomedical Engineering, Hebei University of Technology, Tianjin, China; ^7^ School of Engineering, Saveetha University, Chennai, India; ^8^ Program in Materials, UFPI, Teresina, Brazil; ^9^ Division of Pre-College and Undergraduate Studies, Brown University, Providence, RI, United States; ^10^ School of Pharmacy, Al – Karim University, Katihar, Bihar, India; ^11^ Centre for Global Health Research, Saveetha Institute of Medical and Technical Sciences, Chennai, Tamil Nadu, India; ^12^ Center for Advanced Materials, Qatar University, Doha, Qatar

**Keywords:** ocular drug delivery, nanomedicine, lipid nanoparticles, solid lipid nanoparticles, nanostructured lipid carriers, cationic nanostructured lipid carriers, ophthalmic drug delivery

## Abstract

Ocular drug delivery presents significant challenges due to intricate anatomy and the various barriers (corneal, tear, conjunctival, blood-aqueous, blood-retinal, and degradative enzymes) within the eye. Lipid-based nanoparticles (LNPs) have emerged as promising carriers for ocular drug delivery due to their ability to enhance drug solubility, improve bioavailability, and provide sustained release. LNPs, particularly solid lipid nanoparticles (SLNs), nanostructured lipid carriers (NLCs), and cationic nanostructured lipid carriers (CNLCs), have emerged as promising solutions for enhancing ocular drug delivery. This review provides a comprehensive summary of lipid nanoparticle-based drug delivery systems, emphasizing their biocompatibility and efficiency in ocular applications. We evaluated research and review articles sourced from databases such as Google Scholar, TandFonline, SpringerLink, and ScienceDirect, focusing on studies published between 2013 and 2023. The review discusses the materials and methodologies employed in the preparation of SLNs, NLCs, and CNLCs, focusing on their application as proficient carriers for ocular drug delivery. CNLCs, in particular, demonstrate superior effectiveness attributed due to their electrostatic bioadhesion to ocular tissues, enhancing drug delivery. However, continued research efforts are essential to further optimize CNLC formulations and validate their clinical utility, ensuring advancements in ocular drug delivery technology for improved patient outcomes.

## Graphical Abstract

**Figure:**
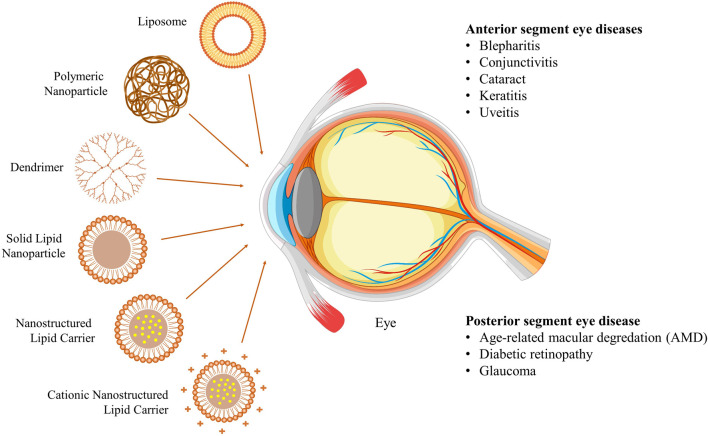
Image courtesy of brgfx, Freepik (https://www.freepik.com/free-vector/human-eye-with-disease_22744188.htm#query=eye diagram&position=13&from_view=search&track=ais&uuid=bb73bb73-11ad-40ef-a565-d6929fd24bda).

## 1 Introduction

**FIGURE 1:**
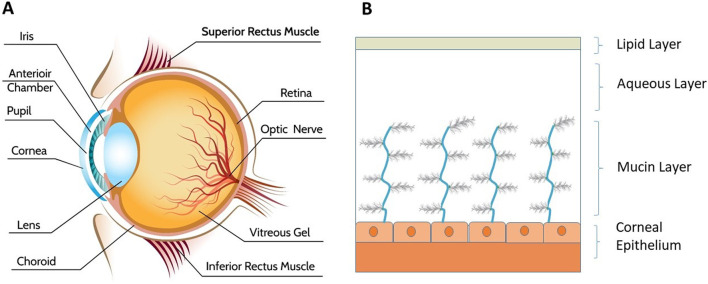
Visually represents **(A)** the anatomy of the human eye [image courtesy of Freepik (Macrovector, 2020)], and **(B)** the layers of tear film essential for ocular health and function.

The human eye plays a crucial role in the body by gathering light signals through photoreceptors and transmitting them to the brain via neuronal signals. The eye is divided into three parts: anterior, posterior, and vitreous, as shown in Figure 1A. The anterior chamber comprises the cornea, iris, ciliary body, and lens (Farid et al., 2017; Ahmed et al., 2023). Specifically, the ciliary body drains waste from the lens and cornea (Suri et al., 2020). The posterior region includes the choroid, neural retina, and retinal pigment epithelium (RPE). Within the vitreous body, the ciliary body continuously produces aqueous humor, a jelly like and transparent substance that helps maintain the shape of the eyeball. Three primary layers, the fibrous layer, the vascular layer, and the neural layer, are present in the eyes. The fibrous layer encompasses the cornea, sclera, and conjunctiva, with the conjunctiva being a thin, mucus-secreting, and well-vascularized tissue (Gukasyan et al., 2008; Garcin et al., 2020) layer responsible for ocular protection and lubrication. The vascular layer is involved in maintaining the blood-ocular barrier, which regulates the passage of substances from the blood into the eye. Finally, the neural layer is located at the posterior part of the eye and is closely associated with the retina.

Liquid solutions are the single most commonly used conventional dosage form for ocular ailments. After topical instillation of eye drops, less than five percent of the dose enters the eye and is absorbed (Loftsson and Stefánsson, 2022). Although they are easy to administer topically (Mandal et al., 2019), they suffer disadvantages such as spillage of the drug because of lacrimal fluid secretion; hence, they have poor bioavailability. Compared with the drug solutions, the suspended particles showed better retention time in the precorneal segment and more contact time. TobraDex® (Scoper et al., 2008) is a widely used ocular suspension of tobramycin (0.3%) and the steroid dexamethasone (Rizzo et al., 2022). Ophthalmic ointments are semisolid dosage forms composed of a mixture of hydrocarbon bases that melt at an ocular temperature of 34°C but the adverse effects associated with it are ocular irritation, interference with vision systemic complications on chronic administration (Patel et al., 2013; Mazet et al., 2020).

The bioavailability of the drug instilled as a conventional dosage form is reduced due to the eye’s anatomical factors, including its limited volume capacity (30 μL) within the cul-de-sac in humans (Ahmed et al., 2023; Awwad et al., 2017; Downie et al., 2021). Drug residence time is also affected by lacrimation and blinking (Sánchez-López et al., 2017b; Akhter et al., 2022). The primary ocular barriers to the anterior portion of the eye for drug delivery are as follows: i) anatomical/static barriers, such as the cornea, conjunctiva and blood-aqueous barrier; ii) physiological/dynamic barriers, such as tear drainage and conjunctival blood flow; and iii) metabolic barriers. The cornea is the main static barrier that prevents the entry of water-soluble drugs (Ahmed et al., 2023; Awwad et al., 2017; Wang and Zhang, 2023). The drug can cross this barrier through transcellular and paracellular transport mechanisms. Intracellular transport is generally followed by lipophilic substances, while the paracellular pathway is followed by hydrophilic drugs. The conjunctival barrier is yet another static barrier that allows hydrophilic molecules to permeate through the conjunctiva while restricting the transport of molecular weight molecules greater than 20 kDa (Gukasyan et al., 2008; Rozi and Mohmad Sabere, 2021). Dynamic barriers involve the drainage of instilled medicine because of tear flow. The instilled drug is also diluted because of flow, thereby reducing the effective drug concentration. Blinking of eyelids also contributes to the overuse of medicine. The tear film provides irrigation and lubrication to the ocular surface. It also provides a protective barrier from foreign particles to the ocular surface. Tears also confer protection against microbes. The tear film consists of three layers, as shown in Figure 1B. Topically instilled drugs have limited bioavailability (<5%) because of the tear film and other physical and biochemical barriers (Awwad et al., 2017). Initially, the lipid stratum serves to inhibit evaporation. The subsequent central layer is referred to as the aqueous or lacrimal stratum. Finally, the mucin stratum emerges. Mucins are glycoproteins that support various structures (Patel et al., 2013; Dam and Brewer, 2023). The metabolic barrier involves the metabolism that occurs in ocular tissues. This metabolism of drugs is catalyzed by cytochrome P-450 reductase and esterase enzymes in the conjunctiva (Sánchez-López et al., 2017b; Patel et al., 2023). Topical ocular instillation is easy to administer, has rapid onset of action, is noninvasive and avoids systemic toxicity. However, the major challenges of low bioavailability associated with this most acceptable route of administration involve the loss of instilled drug solutions from the precorneal zone and metabolic barriers, as mentioned earlier. Addressing these challenges necessitates innovative formulations and delivery strategies aimed at enhancing drug retention and overcoming ocular metabolic barriers, thereby improving the bioavailability and therapeutic outcomes of ocular drug therapies. Hence, there is a need to enhance the bioavailability of topically instilled drugs through different strategies, thereby increasing the effectiveness of ocular pharmacotherapies. LNPs are biocompatible, versatile and mucoadhesive hence these are better than conventional dosage form (Battaglia et al., 2016). The drug loaded LNPs showed three-fold increase in the cumulative amount permeated trough excised cornea of Albino rabbits when compared with the with an aqueous dispersion of the drug. The enhancement of trans-corneal permeation is attributed to bioadhesion over corneal surface because of positive charge of LNPs (Alhakamy et al., 2022). The obvious enhancement in the permeation parameters of the optimal formulation could be ascribed to the positively charged surface of its nanoparticles, which could enable them to interact and integrate with the corneal membrane and lead to better permeation. These system can be used for delivery of drug to retina (Wang and Zhang, 2023). Ocularly, LNP based delivery systems are potential materials for overcoming such barriers.

This review will examine the utilization of LNPs for ocular delivery across the following dimensions. First, the review will cover the fabrication of biocompatible LNPs including SLNs and NLCs as promising drug carriers for ocular delivery. Second, it will discuss the emergence of CNLCs which aids electrostatic bioadhesion to ocular tissue that could lead to efficient and controlled ocular therapy.

Around 100 relevant articles were analyzed to compile this review, providing a detailed insight into the advancements in LNP-based ocular drug delivery systems. The aim of this scoping review is to consolidate existing knowledge, identify gaps, and promote advancement in the manufacturing scalability and innovation in ocular drug delivery using LNP.

## 2 Methods

A comprehensive literature search was conducted to gather relevant studies on the use of SLNs, NLCs, CNLCs for ocular drug delivery from year 2013–2023. To conduct rigorous and credible research, we adopted necessary steps including the initial screening, full text screening, data extraction and data synthesis in the process of study selection and data mining which ensures that the findings are based on a comprehensive review of relevant literature (Srivastava et al., 2023; Srivastava et al., 2023). The databases searched included TandFonline, SpringerLink, and ScienceDirect. The search was restricted to articles published up to December 2023. Keywords used in the search query as (ocular drug delivery OR ophthalmic drug delivery) AND (“solid lipid nanoparticles” OR “Nanostructured lipid carriers” OR “SLN” OR “NLC” OR “CLNC”). Studies were included if they: i) discussed the use of SLNs, NLCs, or CNLCs specifically for ocular drug delivery, ii) provided detailed information on the materials and methods used for NP preparation, iii) presented empirical data on the efficacy of these NPs in enhancing drug delivery to ocular tissues and iv) were published in the English language. Studies were excluded if they: i) focused on non-ocular drug delivery systems, ii) lacked experimental data or were purely theoretical, and iii) had any duplicated studies.

Relevant data were extracted from the selected/representative studies, including:

1. Type of NPs used (SLNs, NLCs, CNLCs).
2. Materials utilized in the formulation development of NPs.
3. Preparation methods employed for these formulations.
4. Experimental models (*in vitro, in vivo*).
5. Outcomes related to drug encapsulation efficiency, drug release profiles, ocular tissue penetration, and therapeutic efficacy.

## 3 Results

The search of database yielded results through various databases. There were 268 results for ‘tandfonline’, 321 for ‘ScienceDirect’ and 1,166 for ‘SpringerLink’. The results were analyzed and around 100 were considered. The extracted data were systematically analyzed to compare the preparation methods, materials used, and the resultant properties of the SLNs, NLCs, and CNLCs. The analysis focused on:

• Identifying trends in the choice of lipid materials and surfactants.
• Evaluating the effectiveness of different preparation techniques (e.g., high-pressure homogenization, solvent evaporation).
• Comparing different types of formulations (SLN, NLC and CNLS) with respect to material, manufacturing and efficacy.

## 4 Novel ocular drug delivery

Various nanoparticulate-based ocular drugs (Nassiri Koopaei and Abdollahi, 2016), including lipid-based NPs, liposomes, niosomes, polymer-based micelles, and dendrimers, have been used for delivery.

### 4.1 Liposomes

Liposome phospholipid vesicles have been used for targeted drug delivery. They can release drugs in a controlled fashion. Lipophilic drugs can be entrapped inside the cavity for ocular delivery (Tasharrofi et al., 2022). A major challenge associated with its formulation is its fabrication and stability. The properties of liposomes can substantially vary with size, lipid composition and surface charge. The corneal penetration of the drug could be enhanced by liposomes through epithelial cell membranes (Csorba et al., 2023).

### 4.2 Dendrimers

Dendrimers are branched polymeric macromolecular compounds. The geometry of the molecule and its size, weight, and surface charge properties must be considered when selecting it as a drug delivery system. It has been used for delivering drugs to the eye (Wang et al., 2021). It possesses bioadhesive properties; hence, it can improve the precorneal retention time. The problems associated with such a drug delivery system include the formation of a veil in the corneal area and blurred vision, which may cause blindness (Sahoo et al., 2008; Gaudana et al., 2009). However, other novel *in situ* gels could be used for ocular drug delivery. They consist of polymeric hydrogels that change from sol to gel due to external factors such as temperature changes, pH changes or ionic strength changes (Suri et al., 2020).

### 4.3 Polymeric nanoparticles

These are finely divided solid particles below less than the size of 1,000 nm. They can be explored for use as a drug delivery system owing to their mucoadhesive character. Drugs are added to the matrix of the nanoparticulate material. Basically, there are two types of NPs: LNPs and polymeric NPs. Polymers such as Eudragit RL, poly (acrylic acid), polystyrene, and Eudragit RS have been used (Mirzaeei et al., 2022). However, other important polymers for ocular drug delivery include poly (dl-lactide-co-glycolide) (PLGA). Sparfloxacin-loaded PLGA NPs have been developed and evaluated for ocular drug delivery (Gupta et al., 2010).

### 4.4 Lipid-based nanoparticles

**FIGURE 2:**
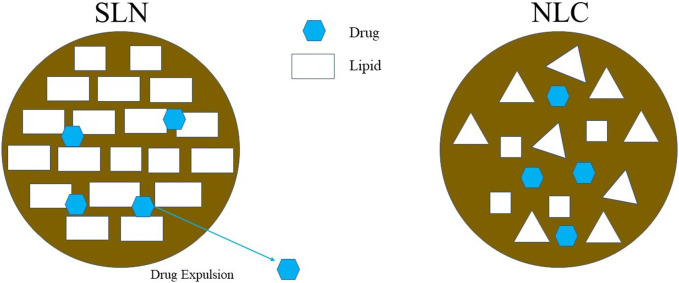
Illustrates the structural differences between SLN and NLC whereas drug expulsion can occur from SLN because of its perfect crystal whereas NLC have better drug loading capacity due to its irregular crystal structure.

These are dispersed colloidal systems in which colloidal particles are dispersed throughout the liquid vehicle (Khan M. S. et al., 2023). The benefit of SLNs as a drug delivery system is that they provide a large surface area and nanosize and improve drug entrapment (Baig et al., 2016). They are prepared using biodegradable and compatible lipid materials such as fatty acids and waxes. Stabilizers, surfactants, and cosurfactants are added to stabilize the mixture. NLCs are prepared by modifying SLNs by mixing liquid lipids with solid lipids (Khan S. et al., 2023). Hence, LNPs can be classified as SLNs or NLCs depending on the inclusion of liquid lipids during their fabrication (López et al., 2023). LNPs evolved from the concept of nanoemulsions, where the oleaginous phase has been replaced by solid lipids, thereby reducing the requirement of high surfactant concentrations to stabilize the system. The major problem associated with these systems is drug expulsion from the particles (Farid et al., 2017) due to the crystallization of solid lipids and poor loading capacity (Figure 2). Compared with their SLN counterparts, NLCs have been developed as advanced versions of LNPs that contain liquid lipids in their matrix, increasing their loading capacity. Along with solid lipids, NLCs are lipid-based nanoparticulate systems manufactured from liquid lipids (Beloqui et al., 2016; Salvi and Pawar, 2019). This system is better than SLNs because of its drug loading capacity (Viegas et al., 2023).

### 4.5 Cationic nanostructured lipid carriers (CNLCs)

**FIGURE 3:**
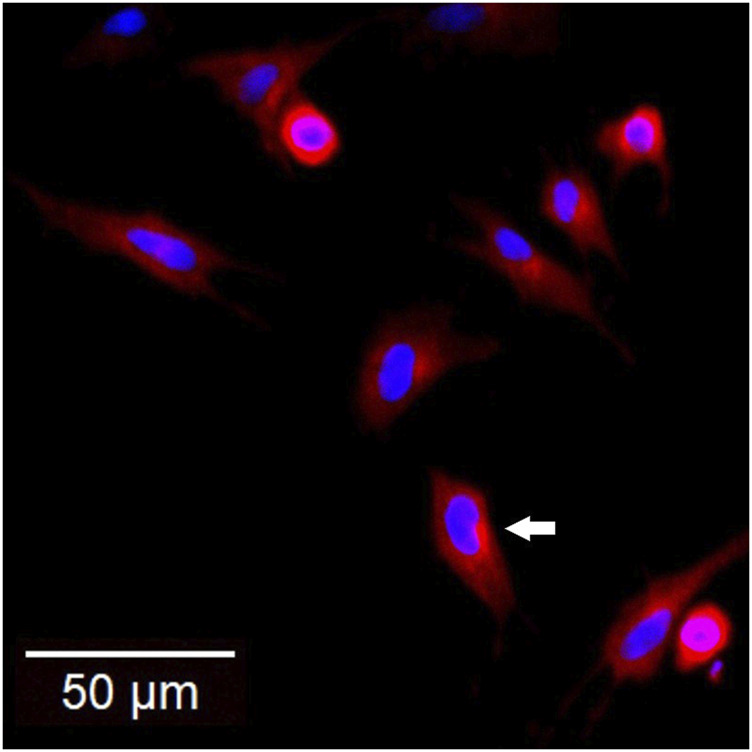
Confocal scanning laser microscopy image showing the uptake of the rhodamine-loaded CNLC formulation (red) into a 2D conjunctival tissue model where the cell nuclei were stained with Hoechst dye (blue). [Adapted from our published work (Baig and Siddiqui, 2020)].

CNLCs are an advanced version of SLNs. They carry a positive charge on the surface and lead to the formation of liquid lipids in their matrix; hence, they are electrostatically attracted to conjunctival cell membranes, which are negatively charged (Liu et al., 2016; Baig et al., 2020). The surface has a positive charge and provides bioadhesion, while liquid lipids are responsible for improved drug loading (Botto et al., 2017). These CNLC features, in addition to their nanoscale size, make them suitable ocular drug carrier systems. The bioadhesion of NPs could increase the retention time in ocular tissue while reducing precorneal drainage after ocular instillation. Ocular irritation could be correlated with cell viability via MTT assays (Hassanzadeh et al., 2017). Formulating CNLC for ocular instillation has improved the bioavailability of the drug (Liu et al., 2016). Studies have demonstrated the effect of zeta potential on rhodamine-loaded LNP formulations (Baig and Siddiqui, 2020) on cell uptake and permeation in cell culture-based ocular models using confocal laser microscopy (Figure 3).

### 4.6 Benefits of novel over conventional ocular formulations

#### 4.6.1 Conventional ocular formulations

##### 4.6.1.1 Merit

• Easy topical administration (Mandal et al., 2019).
• Better retention time in precorneal segment (Loftsson and Stefánsson, 2022).
• Slow release (Patel et al., 2013).

##### 4.6.1.2 Demerit

• Poor bioavailability (Loftsson and Stefánsson, 2022).
• Spillage due to lacrimal fluid secretion.
• Harmful additives (Ahmed et al., 2023).
• Ocular irritation and redness (Patel et al., 2013).

#### 4.6.2 Novel ocular formulations

##### 4.6.2.1 Merit

• Targeted drug delivery (Tasharrofi et al., 2022).
• Enhanced precorneal retention time (Wang et al., 2021).
• Improved bioavailability (Liu et al., 2016).

##### 4.6.2.2 Demerit

• Fabrication and stability challenges (Tasharrofi et al., 2022).
• Corneal veil formation, blurred vision (SAHOO et al., 2008; Gaudana et al., 2009).
• Drug expulsion, poor loading capacity (Farid et al., 2017).

## 5 Advantages of novel ocular drug delivery system

The physicochemical properties of lipid nanocarriers are similar to those of the tear film (Sánchez-López et al., 2017a). This interaction plays an important role in the affinity of LNPs for the ocular surface. This helps to improve the time of residence for LNPs in the conjunctival sac. This process creates a reservoir for the slow release of drugs in the eye. Minimization in dose-frequency has been achieved, which is expected to improve patient compliance with medication. Hence, LNPs are very useful for i) precorneal drug retention, ii) superior segment (retinal) drug delivery (Bonilla et al., 2021; Battaglia et al., 2016), iii) controlled delivery of drugs, iii) patient compliance, and iv) prevention of systemic side effects because the delivery system is effective at low concentrations. For dendrimers, ∼0.03% of the injected dose reaches ∼100 µg of retina/choroid complex in 72 h, amounting to a ∼0.1 mg/g concentration at the target site, which is 100 times greater than the free drug concentration administered via local, intravitreal delivery (Kannan et al., 2023). A parameter that could increase patient compliance is that the drug delivery system should be comfortable for the patient.

## 6 Research and applications of LNPs for ocular drug delivery: a case study

SLNs have been formulated to administer tobramycin to the eyes of rabbits for ocular purposes (Cavalli et al., 2002). Tobramycin containing SLNs was instilled in the eyes of the rabbits, and an improvement in bioavailability was observed in the aqueous humor of the eyes of the rabbits compared to that of a tobramycin solution at similar concentrations. Diclofenac-loaded SLNs were generated using phospholipids and goat lipids (*Capra hircus*) (Attama et al., 2008). A high drug entrapment efficiency of up to 90% was observed for different batches. Compared with those of the placebo particles, the drug-loaded particles showed a high zeta potential. A comparatively less crystalline structure was observed for the particles containing phospholipids.

NLCs have been developed for ocular drug delivery (Shen et al., 2009). Mucoadhesive properties were induced in NLCs using cysteine-polyethylene glycol. A melt-emulsification methodology was employed to prepare cyclosporine-A-loaded NLCs. The mucoadhesive properties of the formulation were evaluated using the mucin particle method. The precorneal retention time also increased. Triamcinolone acetonide-loaded NLCs were developed for intravitreal targeting through the topical administration of colloids (Araújo et al., 2010). High-pressure (up to 600 bar) homogenization was employed for NLC production. A particle size of <200 nm was generated, and ocular toxicity was not observed when the Draize test was used.

Baicalin-loaded SLNs have also been prepared for ocular drug delivery (Liu et al., 2011). An ultrasonication emulsification technique was used to prepare the NPs. The drug permeability of the formulation through the cornea was tested. An ocular irritation study was performed using rabbits. Clotrimazole-loaded SLNs and NLCs were fabricated for ocular use (Das et al., 2012). SLNs demonstrated high drug loading but low release, whereas NLCs showed rapid drug release at low drug loading. Compared with SLN, NLC had a better encapsulation capacity (96% and 99%, respectively) (Makoni et al., 2019). Gatifloxacin-loaded cationic SLNs were prepared for ocular drug delivery (Abul Kalam et al., 2013).

CNLCs (Fangueiro et al., 2014a) with a positive charge on the surface of NPs have been developed through multiple emulsion techniques. The cytotoxicity of CTAB-containing colloidal lipid nanoparticulate dispersions was studied using the human retinoblastoma cell line Y-79. Epigallocatechin gallate (EGCG)-loaded LNPs were prepared using multiple emulsion techniques (Fangueiro et al., 2014b). The bioadhesion/mucoadhesion of the ocular surface was enhanced through the induction of electrostatic attractive forces using cetyltrimethylammonium bromide (CTAB). The particle size was less than 300 nm, which was good for ocular administration. Melatonin-loaded cationic SLNs were developed to improve ocular hypotensive effects (Leonardi et al., 2015). A positive zeta potential was induced on SLN by adding dodecyl-dimethyl-ammonium bromide. Softisan100 was used as the solid lipid control. Ocular tolerability of the nanoparticulate dispersion was also studied *in vivo* on the ocular surface, and the dispersion was found to have good tolerability.

Levofloxacin-loaded stearic acid SLNs were prepared for ocular drug delivery (Baig et al., 2016). The effects of the proportion of surfactant lipids and cosurfactant on the encapsulation efficiency and particle size were studied as dependent variables. Optimization constraints were selected for minimizing the particle size while maximizing the encapsulation efficiency. Indomethacin-loaded NLCs and SLNs were prepared for ocular delivery (Balguri et al., 2016). Chitosan chloride and penetration enhancers were used to enhance transmembrane penetration. The particle size reached 265 nm, whereas + 1.0 mV was the maximum zeta potential and +12.0 mV was the minimum. The entrapment efficiency reached 99%. Good permeation was observed *in vitro* when scleral membranes mounted on Valia-Chien cells were used. A higher concentration of the drug was observed for the NLC formulation *in vivo* than for the other formulations when evaluated in New Zealand White albino rabbits.

Curcumin-loaded NLCs have also been fabricated (Lakhani et al., 2018). A central composite design has been employed for the optimization of physicochemical properties. A hot-melt emulsification method followed by sonication was employed to prepare the NPs. The NLCs demonstrated enhanced permeation of curcumin through excised corneas from Albino New Zealand rabbits when mounted vertically on modified Franz diffusion cells. Besifloxacin HCl-loaded CNLCs were prepared and optimized using design expert software for determining the zeta potential of cationic NLCs (Baig et al., 2020). Cytotoxicity was evaluated through an MTT assay using a conjunctival fibroblast culture-based model. Ocular permeation was studied using a 3D cell culture model.

Clarithromycin-loaded SLNs was also formulated (Nair et al., 2021) using stearic acid for ocular therapy. Tween 80 was used as an emulsifier. A fractional factorial design was used for optimizing the particle size, polydispersity, and percent entrapment efficiency. Lactoferrin-loaded NLCs were prepared for the treatment of Keratoconus, a degenerative disorder (Varela-Fernández et al., 2022). NLCs were fabricated using solid lipids such as glyceryl behenate, glycerol monostearate, and polyxamer as emulsifiers. Double emulsification with the solvent-evaporation technique was used to prepare NLCs. The average particle size of the lactoferrin-loaded NLCs was less than 150 nm, whereas the polydispersity index (PDI) was 0.3, and the zeta potential was −18 mV. Econazole-loaded SLNs were prepared (using the microemulsion method) and modified with positive and negative charges on their surface (Liang et al., 2023). The zeta potentials of the formulations ranged from +19.13 mV to −27.40 mV. The drug-loaded SLNs exhibited sustained release, with less than 20% release in the SLN form compared to 100% release in suspension after 8 h (Liang et al., 2023). Compared with their negatively charged counterparts, the positively charged NPs demonstrated better corneal penetrability.

The role of LNPs as innovative solutions for improving ocular drug delivery is evident because of their controlled release and bioadhesion properties. SLNs and NLCs represent significant progress in ocular drug delivery by employing a positive zeta potential of +20 mV to enhance ocular barrier penetration (Liang et al., 2023). These types of LNPs offer several advantages, such as improved biocompatibility and small size (equivalent to 400 to 600 Da) for ocular barrier penetration (Giri et al., 2023). LNPs offer a means to enhance drug bioavailability while providing controlled drug release. Numerous studies have demonstrated the effectiveness of SLN and NLC for delivering a wide range of drugs to the eye.

## 7 Manufacturing of LNPs for ocular delivery

The primary ingredients that are necessary for making SLNs/NLCs include solid lipids, liquid lipids and emulsifiers. The solid lipids included trigliceride (Dynasan), a mixture of mono-/di-/triglyceride (Imwitor, Compritol, Precirol), triterpenes (Squalene), and waxes (stearic acid, cetyl palmitate), which have been used for formulating SLNs/NLCs for ocular applications (highlighted in Table 1). These ingredients are composed of physiological materials and belong to the generally recognized as safe (GRAS) excipient list. These solid lipids melt above body temperatures, generally exhibiting a melting point above 40°C. The liquid lipids used to fabricate NLCs include fatty acids (oleic acid) and triglycerides (miglyol), such as fatty acids, triglycerides, diglycerides, monoglycerides, steroids and waxes (see Table 1). The selection of solids/liquids is determined by the solubility and compatibility of the drug molecule to be incorporated into the matrix. Liquid lipids disrupt the crustal structure of solid lipids, thereby reducing the chance of drug expulsion and increasing the drug loading capacity. The surfactants used in the formulation include polysorbates (Tween), polyethylene glycols [PEG-32 (Gelucire), polyethylene glycol PEG-8 (Labrasol)], etc.

**TABLE 1:** Novel excipients for the manufacturing of stable LNPs and NLCs for ocular delivery.

Ingredients	Reference
Solid Lipid	
Compritol 888 ATO (Glyceryl dibehenate)	Abul Kalam et al. (2013), Chaudhari et al. (2021), Balguri et al. (2016), Seyfoddin and Al-Kassas (2013), Varela-Fernández et al.( 2022)
Imwitor 900 K	Hao et al. (2011), Almeida et al. (2016), Sakellari et al. (2021)
Glyceryl monostearate	Nair et al. (2021), Varela-Fernández et al. (2022)
Precifac® ATO 5 (Glyceryl distearate)	(Shen et al., 2009; Gonzalez-Mira et al., 2011)
Precirol ATO 5 (Glyceryl palmitostearate)	(Almeida et al., 2016; Abul Kalam et al., 2013)
Stearic Acid	(Leonardi et al., 2015; Seyfoddin and Al-Kassas, 2013; Baig et al., 2016)
Witepsol E85	Almeida et al. (2016)
Gelucire® 43/01	Almeida et al. (2016)
Lipocire® DM	Almeida et al. (2016)
Dynasan	Almeida et al. (2016)
Softisan 142	Almeida et al. (2016)
Cetyl palmitate	Almeida et al. (2016)
• Palmitic acid	Leonardi et al. (2015)
Liquid lipid	
Cremophor EL	Li et al. (2008)
Mygliol® 812	Almeida et al. (2016), Liu et al. (2016)
Labrafac PG	Baig et al. (2020)
Miglyol® 840	Shen et al. (2009), Liu et al. (2016)
Oleic acid	Üstündağ-Okur et al. (2014) Liu et al. (2016), Leonardi et al. (2015)
Softisan 645	Almeida et al. (2016)
Squalene	Chaudhari et al. (2021), Chinthaginjala et al. (2024)
Capryol®	Zwain et al. (2021)
Lauroglycol® 90	Zwain et al. (2021)
Perhidrosqualene	Almeida et al. (2016)
Emulsifier	
Tween 80	Balguri et al. (2016), Gonzalez-Mira et al. (2011), Üstündağ-Okur et al. (2014)
Tween 40	Seyfoddin and Al-Kassas (2013)
Poloxamer 188	Balguri et al. (2016), Abul Kalam et al. (2013), Gokce et al. (2008)
Gelucire® 50/13	Abul Kalam et al. (2013), Baig et al. (2020)
Gelucire® 44/14	Li et al. (2008), Almeida et al. (2016), Liu et al. (2016)
Myrj 52	Liu et al. (2016)
Brij 78 (polyoxyethylene-20-stearyl ether)	Seyfoddin and Al-Kassas (2013)
Labrasol	Guo et al. (2019), Zwain et al. (2021)
Sodium taurocholate	Abul Kalam et al. (2013)

**FIGURE 4:**
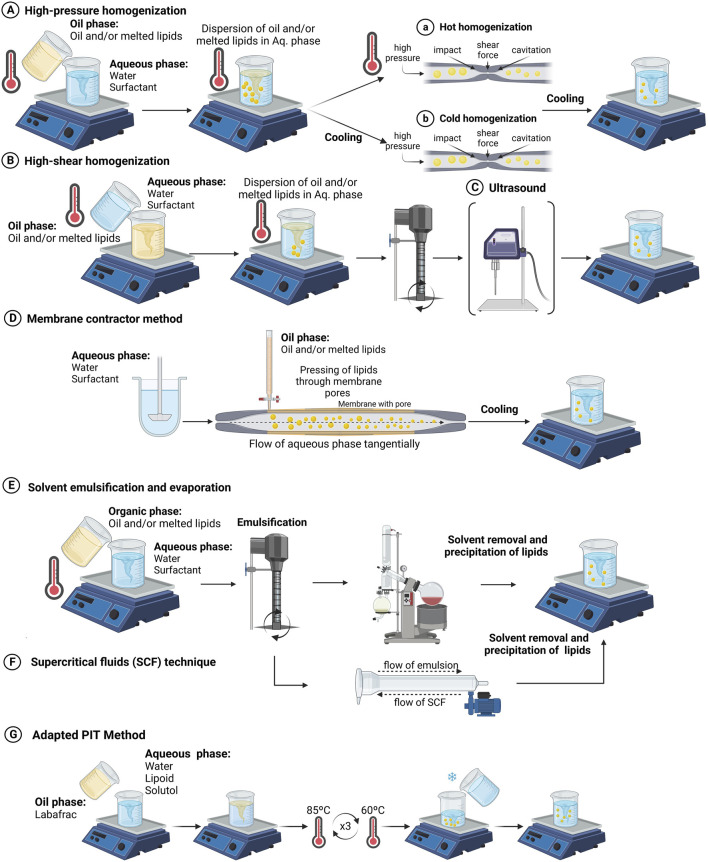
Various methods of preparation of Lipid Nanoparticles (LNPs), including **(A)** high-pressure homogenization, **(B)** high shear homogenization, **(C)** ultrasonication, **(D)** membrane contractor method, **(E)** solvent emulsification and evaporation, **(F)** supercritical fluid technology, **(G)** adapted phase inversion temperature (PIT) method [figure adopted from (Graván et al., 2023)] (*These methods enable the production of LNPs with tailored characteristics for pharmaceutical and biomedical applications*).

**FIGURE 5:**
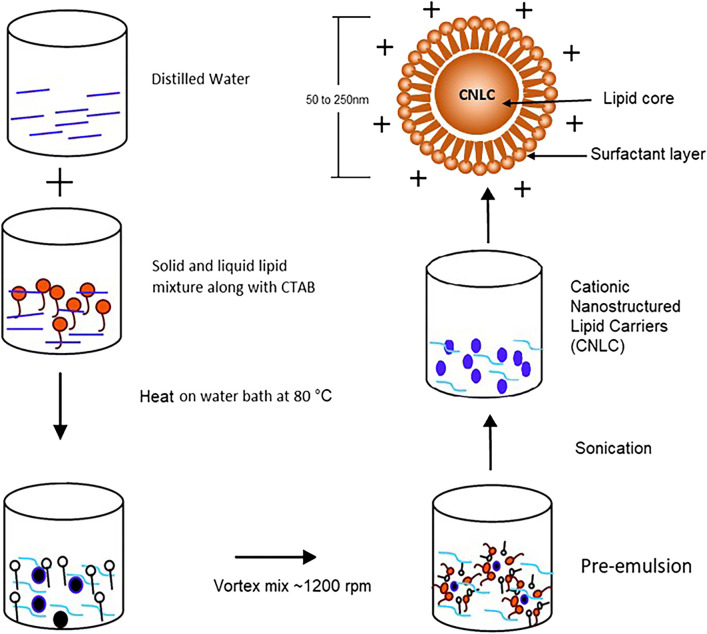
Illustrates the method of preparation for CNLC, highlighting key steps including lipid melting, emulsification, solidification, and characterization with improved drug loading capacity and stability for pharmaceutical applications [figure adopted from (Baig and Siddiqui, 2020)].

A variety of methods have been used for making SLNs/NLCs depending upon the scale of production, particle size, application, etc. Several of these methods for making SLNs/NLCs have been described in the literature (Sastri et al., 2020; Han et al., 2023). The preparation methods are typically based on high-energy and low-energy methods, each with distinct mechanisms and steps (Figure 4). High-energy methods include high-pressure homogenization, which can be performed via hot or cold processes. In hot homogenization, melted lipids and drug are emulsified at high temperatures, followed by high-pressure homogenization and cooling. Conversely, cold homogenization involves rapidly cooling the drug-lipid mixture to form a solid dispersion, followed by high-pressure homogenization. High shear homogenization or high-speed stirring involves dispersing the drug in molten lipids, mixing with a hot aqueous surfactant phase, and homogenizing. Ultrasound is often combined with other techniques to reduce particle size due to its tendency to yield large particles with a wide size distribution. The membrane contractor method passes melted lipids through a membrane under pressure, forming droplets that solidify into SLNs/NLCs upon cooling. The film ultrasonic method involves dissolving lipids in an organic solvent, evaporating to form a film, mixing with water, and sonication to disperse. Solvent emulsification and evaporation techniques dissolve hydrophobic ingredients in an organic solvent, emulsify in an aqueous phase, and evaporate the solvent, causing precipitation. Supercritical fluids extract organic solvents from a lipid emulsion, leading to lipid precipitation with a narrow size distribution. Low-energy methods include the coacervation method, which uses pH changes to precipitate fatty acids from their sodium salt micelles, forming SLNs/NLCs upon cooling. The microemulsion method involves heating lipids and drug, mixing with an aqueous surfactant phase to form a microemulsion, and cooling to solidify the lipids. These diverse methods for making SLNs/NLCs are summarized in Table 2. The CNLCs were prepared using the simple melt-emulsification ultrasonic homogenization method in Figure 5. Post-preparation procedures after the formulation of LNPs, viz. sterilization and freeze drying are then performed (Seyfoddin et al., 2010).

**TABLE 2:** Methods and technologies used for manufacturing of SLN/NLC.

Method	Technology	Instrumentation	Procedure	References
Hot homogenization method	Extrusions, high pressure homogenization	High pressure homogenizer, Extruder, Ultra Turrax® (IKA-Werke, Staufen, Germany)	Under continuous stirring, the lipid is heated above the melting point with the drug mixed with a hot surfactant solution To form a preemulsion, this dispersion is homogenized with a high-shear mixing or extruder machine at temperatures maintained above the melting point of the lipid, then it is subjected to high pressure homogenization in a warm condition. The homogenization processes is repeated until the desired particle size is achieved. The dispersion is cooled at room temperature to obtain a colloidal suspension of lipid NPs	Patil et al. (2015), Ye et al. (2016), Zhuo et al. (2018), Corzo et al. (2020), Chandana et al. (2021), Kataria and Shende (2022), Harish et al. (2023), Dhanya et al. (2023)
Cold homogenization method	Milling, HPH	Ball mill, High pressure homogenizer, Bath sonicator, Ultra Turrax® T25	The drug is dissolved in a molten lipid. This drug-lipid mixture is solidified using dry ice or liquid nitrogen. The solidified mass is milled to powder up to 100 μm particle sizes. The powder so obtained is subjected to the dispersion into a cold aqueous surfactant solution to make presuspension. Finally, the presuspension is subjected to multiple cycles of HPH to make colloidal suspensions of lipid LNPs	Karami et al. (2016), Huynh et al. (2017), Banerjee and Kundu (2018), Sastri et al. (2020), Hernández-Esquivel et al. (2022)
Melt emulsification/microemulsion method	High speed mixing, high shear homogenization	Ultra-Turrax® T25, Ultrasonicator (probe sonicator)	The organic solvent is not used in this method. The lipid is molten above the melting point temperature of the lipid and the drug is solubilized into it. This molten lipid phase is added to a warm aqueous surfactant solution with a high-speed stirring to make o/w microemulsion. It is finally dispersed into cold water assisted with high speed agitation/ultrasonication to form SLN or NLC.	Abul Kalam et al. (2013), Üstündağ-Okur et al. (2014), Balguri et al. (2016), Seyfoddin et al. (2016), Lakhani et al. (2018), Baig and Siddiqui (2020), Chantaburanan et al. (2023)
Solvent emulsification evaporation method (single emulsion)	High speed mixing, high shear homogenization, HPH	Ultra Turrax T-18/10, Ultrasonicator, High pressure homogenizer	A hydrophobic organic solvent is used to dissolve the bulk lipid and aqueous phase to emulsify into a preemulsion through high-speed mixing. Finally, the preemulsion is subjected to ultrasonication or HPH to achieve a desired particle size. The precipitation of the lipid in the residual aqueous phase forms NPs as soon as the organic solvent evaporates	Pooja et al. (2015), Gajra et al. (2015), Baig et al. (2016), Mohammadi-Samani et al. (2018), Faiz et al. (2023)
Solvent emulsification evaporation method (Double emulsion)	High speed mixing, high shear homogenization	Ultra Turrax® Ultrasonicator	The double emulsion method is useful for loading hydrophilic drugs in LNPs. The drug is dissolved in aqueous phase and dispersed in an organic phase using ultrasonication. This primary emulsion (W/O) so generated was added to the aqueous surfactant solution with high shear agitation or ultrasonication to get a water-in-oil-in-water (W/O/W) emulsion. The organic was evaporated with gentle stirring to get a SLN or NLC colloidal suspension	Nabi-Meibodi et al. (2013), Ngwuluka et al. (2017), Aman et al. (2018), Hong et al. (2022)
Solvent emulsification, diffusion method	High shear mixing, high shear homogenization	High speed stirrer (REMI Instruments Ltd., Mumbai, India) Ultra Turrax® (IKA, Staufen, Germany), Ultra-homogenizer (Heidolph Electro, Kelhaim Co., Ltd., Germany)	The drug along with lipid-mix were dissolved in the cosolvent (acetone, ethanol, Ethyl acetate, benzyl alcohol, butyl lactate). The resultant solution is slowly added into an aqueous surfactant solution with high speed stirring followed by ultrasonication. It is at this point that the cosolvents diffuse into the aqueous medium, resulting in the precipitation of lipids and the production of SLN or NLC.	Pardeshi et al. (2013), Leonardi et al. (2014), Ebrahimi et al. (2015), Cynthia Jemima Swarnavalli et al. (2016), Kumar Gupta et al. (2022)
Phase inversion temperature (PIT)	Moderate stirring	Magnetic stirrer, mechanical stirrer	The PIT method involves increasing the temperature of a mixture of surfactants above the phase transition 10°C temperature then quickly cooling it down to room temperature, transform w/o to o/w emulsion. An HLB transforming agent is used to change the phase composition, resulting in a nanoemulsion formation. During the heating stage, mechanical/magnetic stirrers are used to agitate the mixtures continuously	Gao and McClements (2016), Gomes et al. (2017), Ali and Singh (2018), Gomes et al. (2019), Spadaro et al. (2021)
Coacervation	Moderate stirring	Magnetic stirrer	During the process of coacervation, a surfactant solution is heated above the *Krafft Point* while stirring it continuously. Subsequently, alkaline salts of fatty acids are precipitated when the pH is reduced by adding acidifying solutions (coacervating solutions) dropwise to the mixture of molten lipids and polymer (PVA) solution, until complete precipitation of lipids was achieved. Afterwards, cooling was done with continuous stirring	Chirio et al. (2014), Peira et al. (2016), Battaglia et al. (2017), Malekpour-Galogahi et al. (2018), Talarico et al. (2021), Kumar Gupta et al. (2022)
Membrane contactor method	Membrane contactor technique	Membrane contactor assembly [stirrer, pump, pressurized vessel for lipid phase, manometer, tubular SPG membranes (SPG technology, Miyazaki, Japan) and Kerasep ceramic membranes (Rhodia Orelis, France)]	In this procedure, an oleaginous phase is pushed through a membrane while keeping the temperature higher than the lipid’s melting point. This leads to the creation of tiny droplets. Simultaneously, an aqueous phase consisting of surfactants moves on the opposite side of the membrane, sweeping off the droplets from the membrane’s surface. Ultimately, the mixture is cooled to produce SLN/NLC.	El-Harati et al. (2006), Charcosset et al. (2006), Souto et al. (2020), Dhiman et al. (2021), Ali and Staufenbiel (2021), Musielak et al. (2022), Chinthaginjala et al. (2024)
Supercritical fuid methods	Supercritical fluid technique	Supercritical fluid assembly [high pressure syringe pump, high pressure expansion vessel, depressurization valve, heater, thermocouple, etc.]	First, o/w emulsion is prepared, and then the organic solvent is extracted using a supercritical fluid. The top-down injection of the emulsion takes place within an extraction column, while supercritical CO_2_ is introduced from the bottom in a countercurrent manner. Lipids precipitate because of the quick and complete removal of the solvent	Yang and Ciftci (2017), Couto et al. 2017), Trucillo and Campardelli (2019), Saad et al. (2020)

## 8 Challenges in commercialization of ocular nanopharmaceuticals

Safety and efficacy are of prime concern while developing LNPs for ocular use. Sterilization of formulation is also challenging whereas membrane filtration is used for this purpose. LNPs can be manufactured in an industrial set up with high shear homogenizers that are commonly accessible. The major challenges are related to scale-up and production of lipid based formulations are reproducibility, and reliability of the employed methodology (Hallan et al., 2021). A multi-component processing line is required for the manufacture of NPs because the process often entails several phases, such as centrifugation, filtration, lyophilization, emulsification, crosslinking, sonication, emulsification, evaporation of organic solvents, homogenization, filtration, and milling. Therefore, it is still difficult to optimize process parameters to attain important quality features in a repeatable manner at the commercial scale, even though small-scale prototypes with well-established characteristics are relatively straightforward to get. However, LNP based ocular products like Ikervis®, or Cequa® are commercially available (Jacob et al., 2022).

## 9 Conclusion and future perspectives

The LNPs represent a significant advancement in ocular drug delivery, overcoming many limitations associated with the conventional dosage forms. These systems have shown potential for treating conditions such as conjunctivitis, glaucoma, and retinal diseases. The current review has highlighted the versatility and efficacy of SLNs, NLCs and CNLCs in enhancing drug bioavailability and retention time in ocular tissues. The unique properties of these NPs, including their biocompatibility, ability to cross ocular barriers, and potential for targeted and sustained drug release, make them promising candidates for future ophthalmic therapies. Due to the small size and high surface area of NPs, these nanoformulations, such as SLNs/NLCs, are popular among formulation scientists for ocular drug delivery. The SLNs/NLCs are helpful for solubilizing hydrophobic drugs in an aqueous solution, thereby allowing delivery of drugs or biological agents through topical or intravitreal routes. Among formulation scientists, SLNs/NLCs are also among the most common delivery systems for anti-vascular endothelial growth factor (VEGF) agents to prevent retinal neovascularization and macular degeneration. SLNs/NLCs are versatile drug carriers with a promising future for drug delivery to the anterior and posterior segments of the eyes. Antibody-coated SLNs/NLCs can also be used for drug targeting. The incorporation of positive charges in CNLCs, in particular, has demonstrated superior bioadhesion and drug delivery efficacy due to electrostatic interactions with ocular tissues. Studies included in this review show that LNPs can significantly enhance the bioavailability of ocular drugs, reduce dosing frequency, and improve patient compliance. These LNPs have the potential to revolutionize ocular pharmacotherapies by improving drug retention, bioavailability, and patient compliance. However, additional investigations in this area of research are needed to determine the full potential of LNPs for the treatment of patients with ocular diseases and conditions. Despite the challenges in large-scale manufacturing and stringent adherence toward sterilization for the successful commercialization of products, commercialized products like Ikervis® and Cequa® demonstrate the feasibility of these advanced delivery systems. Continued research and development in this field hold the promise of more effective and patient-friendly ocular treatments, paving the way for better management of ocular diseases and improved patient compliance.

Future perspective in the current field could focus on the following: (i) the development of more sophisticated and scalable preparation methods for LNPs will be crucial. Techniques that can consistently produce NPs with optimal size, stability, and drug encapsulation efficiency need to be further refined and standardized, (ii) the formulation of targeted drug delivery to specific ocular tissues or cells (Baig et al., 2024). This includes the development of NPs that can bypass the ocular barriers more effectively and deliver drugs directly to the retina or other deep ocular tissues, and (iii) the development of personalized ocular drug delivery systems using LNPs by tailoring the NP formulation to the specific needs of individual patients based on their genetic profile, disease state, and ocular anatomy.

Continued research into optimizing formulation parameters, ensuring biocompatibility, and addressing regulatory considerations will be crucial for advancing the clinical translation of cationic lipid-based nanoparticles in ocular drug delivery, ultimately benefiting patients through improved treatment outcomes and enhanced ocular health management.
